# Clinical Peptidomics in Acute Leukemias: Current Advances and Future Perspectives

**DOI:** 10.1021/acs.jproteome.4c00807

**Published:** 2024-11-18

**Authors:** Danila Felix Coutinho, Túlio Resende Freitas, Ana Carolina Silva Batista, Mariana Torquato Quezado de Magalhães, Adriano de Paula Sabino

**Affiliations:** † Department of Clinical and Toxicological Analyses, Faculty of Pharmacy, Federal University of Minas Gerais, Belo Horizonte, Minas Gerais 31270-901, Brazil; ‡ Department of Biochemistry and Immunology, Institute of Biological Sciences, Federal University of Minas Gerais, Belo Horizonte, Minas Gerais 31270-901, Brazil

**Keywords:** Clinical Peptidomics, Acute Leukemias, Blood Biomarkers

## Abstract

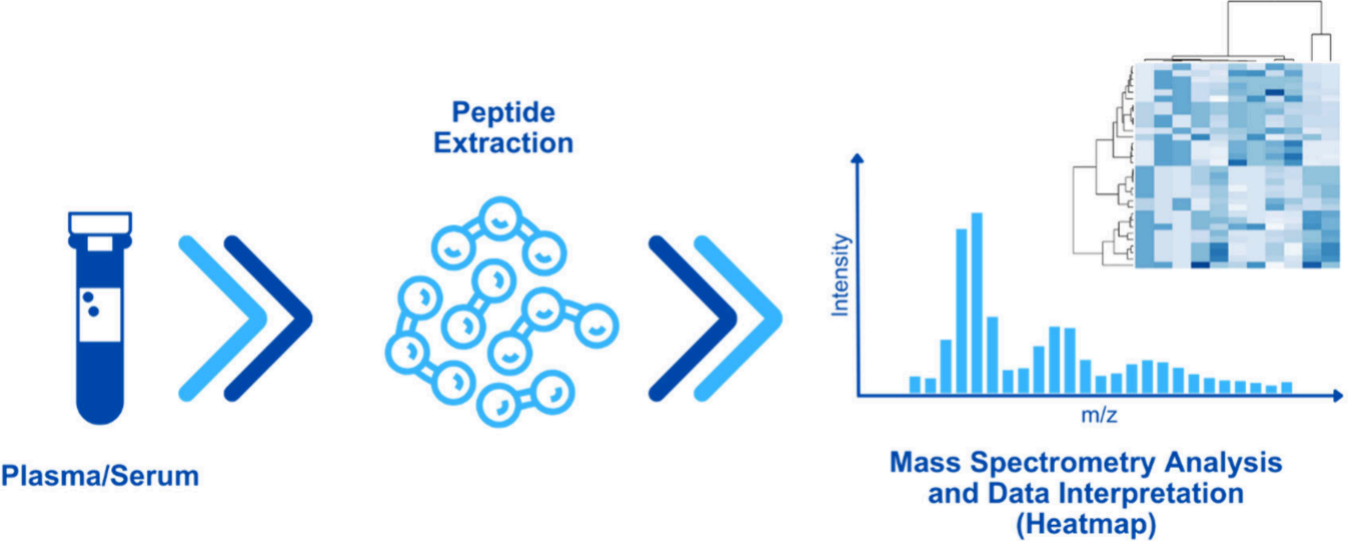

The study of circulating peptides in the blood offers significant opportunities for diagnosing, stratifying, and managing various diseases. With recent technological advances and the ongoing need to understand complex diseases such as acute leukemias (AL), peptidomic analysis of peripheral blood, especially serum and plasma, has become increasingly important for studying human biology and pathophysiology. Here, we provide insights and perspectives on technological developments and potential clinical applications using widely used peptidomic analysis methods. We discuss examples where peptidomics using serum or plasma has contributed to the understanding of AL. Specifically, we highlight the scarcity of peptidomic studies applied to AL and emphasize the importance of exploring this area, as the few published studies present promising results that can significantly contribute to precision medicine.

## Introduction

Acute Leukemias (AL) make up a heterogeneous group of hematological malignancies and are among the most prevalent diseases worldwide. They are divided into Acute Lymphoblastic Leukemia (ALL) and Acute Myeloid Leukemia (AML).^1^ The excessive proliferation of immature leukocytes in the blood and/or bone marrow disrupts the production of functional blood cells, leading to anemia, thrombocytopenia, and immunosuppression.^2^

Diagnosing AL is challenging due to the nonspecific nature of symptoms and the lack of specific biomarkers. These limitations, combined with the complexities of the molecular profiles of AL, not only impair prognostic and treatment strategies but also result in low survival rates, highlighting the need for innovative approaches in precision medicine.^3^

Despite advances, AL diagnosis still relies on invasive methods, such as bone marrow biopsy, which are essential for performing a myelogram. A promising alternative is the use of peripheral blood, which is widely used in laboratories due to its ease of collection and minimally invasive nature. Recently, peripheral blood has been explored in protein studies to identify potential cancer biomarkers. This technique, known as liquid biopsy, aims to monitor pathologies, primarily cancer, using body fluids, including peripheral blood.^4,5^

Proteomics aims to analyze proteins, while Clinical Peptidomics (CP) focuses on the analysis of the low molecular weight proteome (peptides).^6^ These peptides, usually smaller than 20 kDa, reflect the state of a sample or tissue. CP has been employed to identify various activators, inhibitors, and even potential protein substrates^7^ However, despite its significance, studies on the peptidome, which encompasses peptides present in plasma or serum, are still limited.^8^

Mass Spectrometry (MS) has been used in CP, enabling the identification and quantification of peptides in various types of biological materials.^7^ While MS is common in peptidomic studies, its application to plasma peptide analysis is still limited.^9^ CP could serve as a valuable tool in aiding the discovery of potential biomarkers in AL.

This review aims to explore the potential of CP in identifying AL biomarkers, with the goal of optimizing disease management more effectively and improving the quality of life for patients.

### Acute Leukemias: An Overview

**Figure 1:**
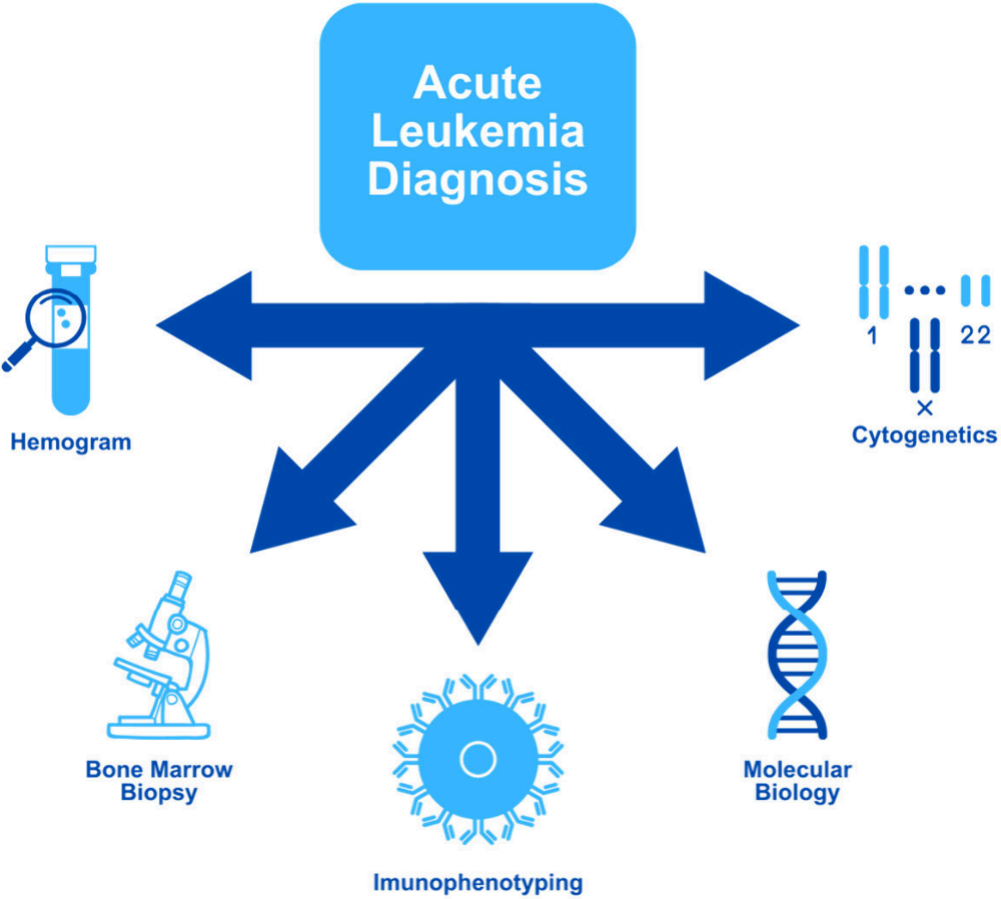
Laboratory tests as diagnostic tools in acute leukemias.

AL are hematological malignancies characterized by the uncontrolled proliferation of immature precursor cells in the bone marrow, disrupting the normal production of blood cells.^10^ The World Health Organization (WHO) primarily classifies these malignancies into AML and ALL, with subtypes defined by cytogenetic, molecular, and morphological criteria (Figure 1).^11^ In the era of precision medicine, molecular profiling has become essential.^12^

The diagnosis of AL relies on gold-standard methodologies including morphological analysis, flow cytometry, cytogenetic analysis, molecular techniques, and bone marrow biopsy. Morphological analysis of leukemic cells, performed on peripheral blood smears and bone marrow aspirates, is an essential initial step for identifying precursor cell abnormalities.^13^ Bone marrow biopsy allows for a direct assessment of cellularity and marrow architecture, which is essential for diagnosing and staging the disease. Flow cytometry identifies AL subtypes through specific surface markers, while cytogenetic analysis, including FISH (Fluorescence in Situ Hybridization),^14^ detects significant chromosomal abnormalities.^15^ Molecular techniques, such as PCR (Polymerase Chain Reaction) and next-generation sequencing, are used to identify mutations and translocations with high prognostic value.^14,16^ Although each technique provides critical information, the definitive diagnosis of AL is based on the combined results of these methodologies, which together offer a comprehensive view of the disease, enabling precise classification, staging, and monitoring throughout treatment.^17^

Treatment for AL typically involves intensive chemotherapy and may include stem cell transplantation. Recently, innovative alternatives have emerged, such as targeted therapies based on the genetic profile of AL. For example, immunotherapy, including Chimeric Antigen Receptor T-cell (CAR-T) therapy, has shown promising results.^1,18^ While these recent advancements represent significant progress, effectively managing AL remains a major challenge, particularly due to the complexity and diversity of patients’ genetic profiles.

### Acute Myeloid Leukemia (AML)

AML is characterized by the disordered clonal proliferation of myeloid precursors in the bone marrow.^1,10,19^ In the United States, the annual incidence of AML is approximately 20,000 cases.^12,20,21^ While the incidence remains relatively constant among children and young adults, there is a significant increase in elderly individuals.^10^ The median age at diagnosis is 68 years, with the highest number of cases occurring between the ages of 65 and 74.^10,12,22^ Additionally, the disease is slightly more common in men than women and more prevalent in individuals of Caucasian descent compared to other ethnic groups.^12^

Genetic abnormalities are found in up to 50–80% of patients with AML, particularly among the elderly.^10^ Common issues include the loss or deletion of chromosomes 5, 7, Y, and 9, as well as specific chromosomal translocations.^10,12^

In most cases, the cause of AML is uncertain, although there is evidence pointing to genetic factors. Additionally, environmental factors, such as exposure to toxic chemicals, have also been associated with the disease.^12^ Individuals previously diagnosed with myelodysplastic syndromes or myeloproliferative neoplasms, or those who have undergone radiation and chemotherapy treatments, are at a higher risk of developing AML.^23^ Hereditary genetic conditions—including Fanconi anemia, Bloom syndrome, Down syndrome, among others—also increase the predisposition to AML.^12,24^

Treatment for newly diagnosed AML traditionally involves induction therapy, followed by consolidation. With the advancement of precision medicine, targeted therapies using FLT3 and IDH1/IDH2 inhibitors have been incorporated for patients with specific mutations in these genes.^25^ In some cases, hematopoietic stem cell transplantation has proven to be beneficial, particularly for patients with high-risk genetic characteristics. However, this option carries its own risks and complications.^12^

In elderly patients, treatment can be more complex due to reduced tolerance for intensive chemotherapy and the presence of comorbidities. Alternatives such as hypomethylating agents (e.g., azacitidine and decitabine) and low-dose cytarabine have been explored, showing varying degrees of efficacy. New approaches, including the use of venetoclax in combination with hypomethylating agents, have demonstrated promising results in improving outcomes for this group.^26^

Recently, oral azacitidine was approved as a maintenance therapy for patients in their first remission, indicating a potential shift in the standard of care. Several other maintenance therapies are currently under clinical investigation, offering an optimiztic outlook for future advancements in AML treatment.^12^ These developments underscore the increasing importance of personalized therapeutic strategies tailored to each patient’s genetic profile.

As our understanding of AML deepens, the patient prognosis has shown improvement over time. Advances in genetic insights and the introduction of novel treatments have significantly increased the precision and efficacy of therapeutic approaches, leading to better patient responses and outcomes.^27,28^ However, challenges remain, and ongoing research is essential to further improve treatment strategies and overcome the genetic heterogeneity associated with AML.

### Acute Lymphoblastic Leukemia (ALL)

In ALL, abnormal differentiation of hematopoietic stem cells leads to an accumulation of dysfunctional lymphoblasts. This results in a reduced count of functional leukocytes and red blood cells, impairing both the immune response and oxygen transport to peripheral tissues.^29^

ALL is more common in children, though it is also observed in adolescents, adults, and, less frequently, in the elderly.^29^ The annual incidence of ALL in children is estimated to be about 3 to 4 cases per 100,000 in developed countries, making it the most common childhood cancer.^30^

Similar to AML, the etiology of ALL has been studied, with studies pointing to environmental exposures, genetic factors, and infections.^1,10,31^ The most widely used classification today is the WHO classification, which subdivides ALL into three main subtypes: unspecified B-cell ALL, B-cell ALL associated with genetic abnormalities, and T-cell ALL. B-cell ALL is the most common form, while T-cell ALL, though less frequent, generally has a poorer prognosis.^32^ Regarding B-cell ALL, there are several characteristic genetic alterations, including intrachromosomal amplification of chromosome 21. A recent classification of B-cell acute lymphoblastic leukemia (B-cell ALL) has unveiled its genetic complexity, subdividing it into 23 distinct subtypes—each with specific prognostic and therapeutic implications.^33^

Biomarkers in ALL are essential for the diagnosis and prognosis of the disease. In developed countries, most cases of ALL present with the ETV6/RUNX1 translocation or a hyperdiploid leukemic clone. However, only 1% of healthy newborns carry cells with t(12;21) [ETV6/RUNX1] translocation. The rate of exposure to infections in developing countries is suggested as a possible reason for the lower incidence of ALL compared with developed countries. Although the exact sequence of events is still unclear, recent studies have suggested the presence of mycoviruses, such as *Aspergillus flavus*, as a potential infectious agent that may trigger ALL.^10^

In the therapeutic context of ALL, conventional treatment is often divided into several phases, including induction, consolidation, and, in some cases, maintenance therapy. Chemotherapeutic agents, such as vincristine and prednisone, are commonly used in the induction phase to achieve complete remission. This is followed by a consolidation regimen that may include hematopoietic stem cell transplantation depending on the severity of the disease and the presence of risk factors.

Recently, targeted therapies and immunotherapies have gained prominence as additional or alternative options, especially for refractory or relapsed cases. These include tyrosine kinase inhibitors (e.g., imatinib for Philadelphia chromosome-positive ALL) and monoclonal antibodies such as blinatumomab and inotuzumab ozogamicin.^34^

### Biomarkers in Acute Leukemias: Advances and Opportunities with Clinical Peptidomics

Biomarkers are measurable indicators within the body that reflect normal biological processes, pathological conditions, or responses to therapeutic interventions, aiding in disease identification and treatment efficacy assessment.^35^ In the context of AL, biomarkers play an essential role in identifying specific subtypes, monitoring disease progression, and evaluating treatment response.

Research into new biomarkers using advanced methodologies has expanded significantly in recent years. Biomarkers can be quantified and play a crucial role in assessing disease progression and therapeutic efficacy.^36^

In AL, these biomarkers are essential for identifying specific subtypes.^36,37^ In AML, for example, in addition to immunophenotyping biomarkers, genes like FLT3, NPM1, and CEBPA, along with chromosomal abnormalities such as t(8;21) or inv(16), have a considerable prognostic impact. Other molecular markers, such as mutations in the IDH1/IDH2 and DNMT3A genes, help predict clinical outcomes and can also be potential targets for targeted therapies. The use of these markers in clinical protocols is important for the advancement of AML treatment, enabling a personalized and optimized approach for patients.^18,20,36,38^

Other biomarkers used in ALL include the immunophenotypic profile, one of the most extensively studied aspects, which enables differentiation between B-cell ALL (B-ALL) and T-cell ALL (T-ALL). Additionally, genetic alterations such as translocations and mutations hold significant diagnostic and prognostic value. For example, the presence of the t(9;22) translocation, which results in the BCR-ABL fusion gene, is a biomarker of poor prognosis and a therapeutic target for tyrosine kinase inhibitors. Other relevant genetic alterations include t(12;21) and t(1;19), which have distinct prognostic implications. Furthermore, the detection of mutations such as NOTCH1 in ALL-T and IKZF1 in ALL-B is also of clinical interest.^30^

Currently, biomarkers play a fundamental role in the diagnosis, prognosis, classification, and personalized treatment of AL, making them highly significant in clinical practice.^38^ Conventional methods—such as flow cytometry, cytogenetic analyses, and molecular techniques—remain the gold standard for diagnosing AL due to their good sensitivity and specificity.^17^ These methods are widely used and are well-established in clinical settings. However, despite their effectiveness, they present limitations within the context of precision medicine as they do not always capture the complex molecular heterogeneity that characterizes leukemias.

Flow cytometry, for instance, detects surface proteins and cell markers with sensitivity higher than that of morphological analysis; however, it does not directly identify specific genetic alterations. This technique also has limitations when tumor cell concentrations are low in samples, compromising early detection, particularly in initial stages of neoplastic infiltration.^39^ Similarly, cytogenetic analyses, while effective at detecting structural and numerical chromosomal abnormalities, have limited resolution for identifying subtle genetic mutations such as small insertions or deletions. Furthermore, the cytogenetic process is complex, time-consuming, and costly, which can delay diagnosis and treatment initiation, underscoring the need for more sensitive and comprehensive methods for the diagnosis and management of acute leukemia.^14^

In this context, MS emerges as a promising alternative. With high sensitivity and specificity, MS enables the rapid identification and quantification of biomarkers while also offering lower operational costs compared to conventional techniques such as flow cytometry, whose reagents, like monoclonal antibodies, are significantly more expensive. Recent technological advances have improved the linear range and ease of use of MS in clinical laboratories without compromising analytical quality.^40,41^ Thus, MS is not only an economically viable long-term option but also holds the potential to deepen our understanding of AL pathogenesis and identify possible therapeutic targets.

MS, as a tool in CP, stands out as an indispensable complementary approach for identifying biomarkers that capture broad molecular changes, often preceding the onset of clinical symptoms. The continuous identification of biomarkers through peptidomics holds the potential to drive precision medicine forward, enhancing the management of AL and contributing to improved clinical outcomes and quality of life for patients.^36^

### Clinical Peptidomics

Peptides play a fundamental role in various biological functions, ranging from intracellular signaling to defending against external pathogens such as bacteria and viruses.^42,43^ The early 1980s marked the beginning of using MS for peptide research.^44^ During that period, peptides were isolated from brain tissues and measured in picomolar concentrations through an innovative technique combining MS with field desorption collision activation.^44^ Since then, there has been considerable advancement in analytical tools for peptide research, including substantial improvements in chromatographic separation, increased sensitivity and accuracy of mass spectrometers, and the development of advanced bioinformatics programs for processing large volumes of data.^45−48^

The term “peptidomics” was first introduced by Schrader^43^ et al. in 2000 and can be defined as the study of endogenous peptides ranging from 2 to 50 amino acids (0.2 to 10 kDa).^42,43,49^ The workflow in CP begins with a biological sample from which peptides are extracted. The extraction process varies, depending on the type of sample and the specific research objectives. Next, these peptides are subjected to MS to acquire spectra. Finally, the obtained data are analyzed using advanced bioinformatics tools.

CP encompasses a broad range of applications, including diagnostics, drug discovery, food science, and more.^5,50,51^ In diagnostics, peptidomics plays a very important role in the early detection and prognosis of diseases by providing sensitive and specific biomarkers for conditions such as cancer and infectious diseases.^5,51^ Through liquid biopsies, peptidomics enhances personalized medicine by efficiently detecting disease-specific physiological changes,^51^ while immunopeptidomics supports the development of cancer therapies by identifying potential therapeutic targets.^52^ In drug discovery, peptidomimetics are designed to replicate peptide structure and function, overcoming limitations such as low metabolic stability and reduced bioavailability.^53,54^ Technological advancements in MS and bioinformatics have further refined peptide detection and analysis, positioning peptidomics as a promising approach for biomarker discovery and therapeutic innovation, despite challenges in clinical application.^55^

### Liquid Biopsy and Peptidomics: Sample Types, Processing, and Storage Strategies

Liquid biopsy uses biomarkers in body fluids for diagnosis, disease monitoring, prognosis, and other applications. The advantage of liquid biopsy is that it provides a less invasive diagnostic option for patients with AL.^56^

Plasma and serum, obtained from whole blood, are valuable for peptidomics studies because they provide comprehensive information about the entire organism.^3^ The process of obtaining serum is different from plasma, as it is allowed to clot naturally without the addition of anticoagulants, taking about 30 min for complete coagulation. During this period, important peptides can be degraded by peptidases present in the sample. This degradation can lead to significant variation in subsequent analyses, affecting the accuracy in measuring potential biomarkers.^57^

Human plasma, in particular, is characterized by its pale yellow color and its ability to keep blood cells in a suspension. The prolonged stability of peptides, often ensured by the use of anticoagulants such as ethylenediaminetetraacetic acid (EDTA) and citrate, makes plasma a preferred choice for peptidomics studies.^57^ This preference is highlighted by Mahboob et al. (2015), who emphasize the importance of plasma in peptide research. Dufresne et al. (2018) identified between 14,000 and 26,000 human proteins in EDTA-treated plasma.

While plasma is a valuable resource for research, its use in CP presents several limitations that can compromise the reliability of biomarker identification. Among these challenges are the complexity of plasma, the presence of abundant proteins that can mask peptides present in low concentrations, endogenous proteolysis, and interference from medications or toxins. Detecting peptides in low concentrations is particularly challenging due to their tendency to degrade.^46^ To minimize these effects, it is essential to adopt rigorous strategies from sample collection to processing. The use of protease inhibitors, chaotropic agents, rapid freezing techniques, or thermal inactivation, along with immediate storage at ultralow temperatures (−80 °C), are critical practices for preserving peptide integrity.^46,58,59^

During processing, it is essential to apply standardized techniques, such as refrigerated centrifugation and specific methods for depleting abundant proteins, to prevent the loss or modification of target peptides. The use of control standards, such as synthetic or internal peptides added to the samples, allows for the monitoring of peptide integrity throughout the entire process, facilitating the detection of potential degradation.

Additionally, the physicochemical properties of plasma can significantly influence the quality and interpretation of peptidome data, requiring extra care in sample handling. Therefore, the implementation of good laboratory practices and the adoption of standardized guidelines are essential to ensure data reliability and to guarantee that the identified biomarkers are clinically relevant for the development of personalized treatments, especially in heterogeneous diseases such as AL.^5^

Another critical challenge is the lack of standardization in sample collection and preparation protocols, which can lead to variability and an increase in the likelihood of inconsistent data. To ensure efficient peptide recovery, the adoption of standardized methods for sample preparation is imperative, resulting in more reliable and reproducible peptidome studies. This need has prompted initiatives such as the Human Proteome Organization (HUPO) to recommend uniform procedures, promoting greater consistency in laboratory practices and, consequently, in results.^3,5,60,61^

### Peptide Extraction Techniques, Ionization Methods, and Applications in Mass Spectrometry

Although it is related to proteomics, CP faces specific challenges. One of these challenges is developing extraction techniques capable of isolating a wide range of peptides, considering factors such as size, polarity, and solubility, while simultaneously excluding interferents like abundant proteins and lipids in peripheral blood.^5^

**Figure 2:**
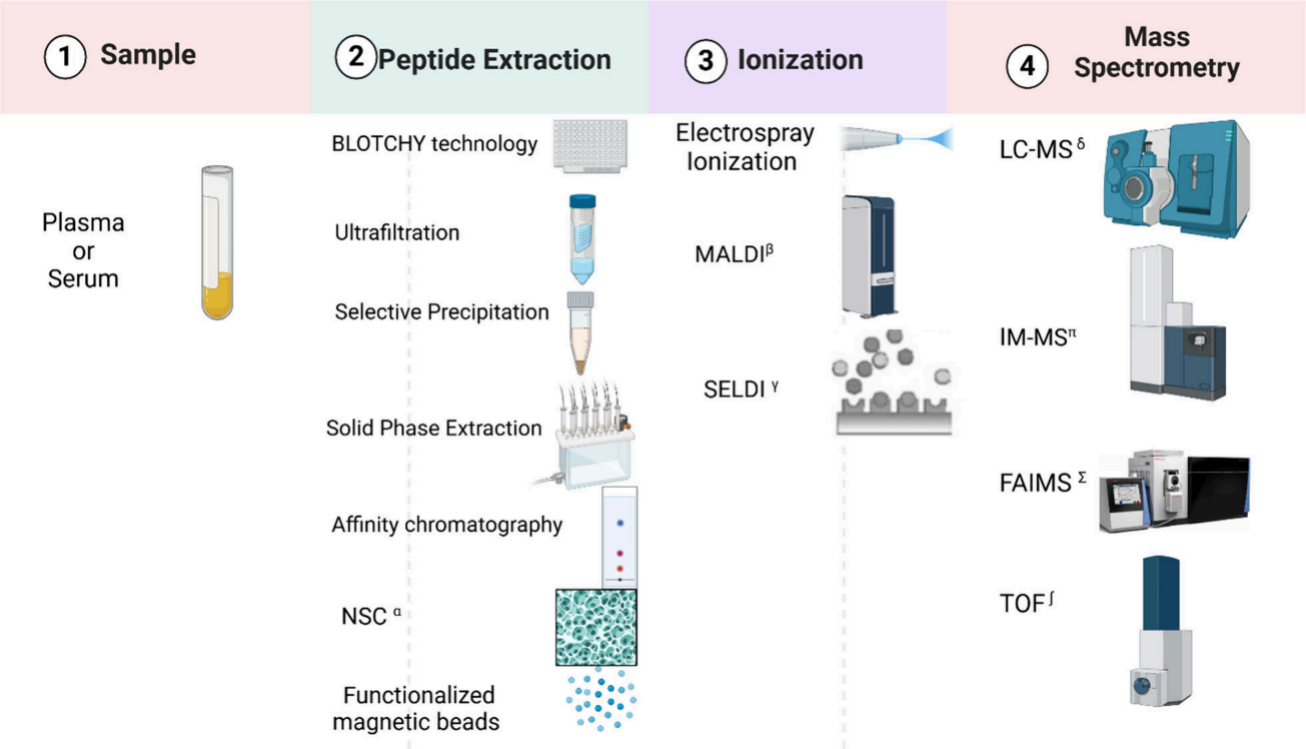
Overview of sample preparation, ionization, and MS techniques for peptide analysis. Made with biorender α NSC-Nanoporous silica chips; β MALDI - Matrix-Assisted Laser Desorption/Ionization; γ SELDI - Surface-Enhanced Laser Desorption/Ionization; δ LC-MS - Liquid Chromatography-Mass Spectrometry; π IM-MS - Ion Mobility-Mass Spectrometry; ∑ FAIMS - High-Field Asymmetric Waveform Ion Mobility Spectrometry; ∫ TOF - Time-of-Flight.

Various techniques are employed to extract peptides from complex biological samples, each with its advantages and disadvantages (Figure 2). When dealing with biological samples like plasma and serum, the presence of abundant proteins must be considered, as they can interfere with peptidomic analyses. To minimize this interference, the BLOTCHIP technology can be used. This method involves the direct transfer of peptides from the gel to a MALDI-TOF/MS analysis plate, allowing for the rapid and efficient analysis of peptides in clinical samples without the need for pretreatments such as the removal of abundant proteins.^49^

Another extraction technique is ultrafiltration, which uses membranes with different molecular weight cutoffs. This technique offers speed, but its effectiveness can be compromised by contamination and the potential partial loss of some peptides.^62,63^ Selective precipitation strategies using organic solvents or acids are also utilized, but suffer from the same limitations of selective losses and peptide aggregation. Solid-Phase Extraction (SPE) columns,^64^ especially Hydrophilic–Lipophilic Balance (HLB) columns^65^ and C18 chromatography resin (zip tip), are widely used to retain analytes and remove interfering compounds.^66^ Alternatives such as affinity chromatography^64^ and the use of functionalized magnetic beads are also employed, with the latter offering opportunities for affinity purification^55,67,68^

Nanoporous Silica Chips (NSCs) represent another extraction method. These specific surfaces selectively capture and protect peptides from enzymatic degradation during the extraction and purification of biological samples.^69^ Each of these methods presents specific challenges, and none offers a universal solution, making the optimization of the extraction process a critical step in peptidomic research.^46,62^

In the context of CP, the diverse physicochemical properties of peptides require ionization techniques to convert substances into ions. When coupled with MS, these techniques significantly enhance the MS efficacy, allowing for more precise and detailed analyses. By transformation of complex peptides from clinical samples into detectable ions, ionization methods improve MS performance. Common ionization methods used in CP include Electrospray Ionization (ESI), Matrix-Assisted Laser Desorption/Ionization (MALDI), and Surface-Enhanced Laser Desorption/Ionization (SELDI).^6^ Accurate mass measurement and tandem fragmentation are ideal for more reliable identification.^5,46,70^

MS techniques are fundamental for detailed peptidomic analyses and are based on measuring the mass-to-charge ratio (*m*/*z*) of the ions. Tandem Mass Spectrometry (MS/MS) can selectively fragment peptide ions and generate detailed fragmentation spectra, which are crucial for the precise determination of peptide amino acid sequences. Ion Mobility-Mass Spectrometry (IM-MS) enhances analysis selectivity by separating ions based on the *m*/*z* ratio, shape, and size, facilitating the distinction between similar molecules. High-Field Asymmetric Waveform Ion Mobility Spectrometry (FAIMS), an ion mobility technique, selectively filters ions based on their mobility properties under an asymmetric electric field, resulting in lower background noise and better resolution.^46,70,71^ Time-of-Flight (TOF) measures the ion mass based on the time it takes for ions to travel a standardized distance. Additionally, Liquid Chromatography-Mass Spectrometry (LC-MS/MS) combines chromatographic separation, which allows for the preseparation of complex sample components, with the detailed analysis provided by MS/MS.^46,71^

The continuous development of more precise and reproducible techniques, along with reduced costs in mass spectrometry, will contribute to a deeper understanding of peptides. This progress, fueled by technological innovation and interdisciplinary collaboration, will thus promote scientific advancement.^51^

Although broad accessibility to peptidomics in clinical settings remains a future goal, the literature highlights efforts to reduce analytical costs. Cost reductions in mass spectrometry involve strategies such as data compression, equipment miniaturization, and computational advancements, focusing on optimizing data storage and dissemination.^72^ The miniaturization of spectrometers allows for more compact and efficient devices,^73^ while advances in Long Short-Term Memory (LSTM) networks enhance spectral detection with reduced resource usage.^74^

In clinical settings, MALDI-TOF is already widely used in microbiology for the rapid identification of microorganisms. Its introduction in clinical microbiology laboratories has not only reduced costs but also improved workflow efficiency by reducing identification times to a 24- to 48 h window and lowering reagent costs. Furthermore, MALDI-TOF eliminates the need for subcultures and microscopy, improving clinical outcomes, shortening hospital stays, and reducing healthcare costs.^75^

These advancements point toward a future where peptidomics could see wider clinical integration, particularly in resource-limited settings. As mass spectrometry technologies, like MALDI-TOF, continue to decrease in cost and increase in efficiency, the detection of peptidomic biomarkers may become an accessible and effective tool for disease diagnosis and monitoring. This progress not only expands diagnostic and therapeutic possibilities but also promotes a more personalized and cost-effective approach with the potential to transform the clinical management of various conditions and significantly improve health outcomes.

### Applications of Clinical Peptidomics in Oncology

CP has the potential to significantly contribute to cancer diagnosis, prognosis, and therapy, as highlighted by Foreman et al. (2021).^5^ A recent example of this application is the study by Xu et al. (2023),^7^ which investigated serous tissue samples from patients with colorectal cancer. Using LC-MS/MS for the extraction and analysis of low molecular weight peptides, the study identified 133 peptides, with 25 showing upregulation and 34 showing downregulation. These changes in peptide abundance suggest their potential as diagnostic and therapeutic biomarkers. This study underscores the importance of peptides in the context of colorectal cancer and highlights the need for future research to validate these findings and explore the specific functions and mechanisms involved more deeply.

In a study conducted by Padoan et al. (2018), which included patients with prostate cancer, the MALDI-TOF technique was used to investigate potential biomarkers. This study identified limitations of the technique regarding reproducibility and analytical variability; however, it is possible to improve the method to address these issues. Despite the criticisms related to variability, the study reinforces the applicability of MALDI-TOF/MS in urological oncology, suggesting that with appropriate statistical approaches, peptidomic data can be adjusted to provide more reliable and accurate information.^6^

Demonstrating the utility of CP in cervical cancer screening, Rungkamoltip et al. (2023) identified distinct peptide patterns between healthy women and those with cervical cancer through MALDI-TOF-MS analysis. These differences in peptide profiles indicate that certain mass-to-charge ratio peaks could serve as potential biomarkers for the early detection of cervical cancer.^76^

The study conducted by Biskup et al. (2017) explored the peptidomics of ascitic fluid from patients with epithelial ovarian cancer, providing relevant information on the N-glycome and its variations. The N-glycome of the ascitic fluid was reported and compared with the N-glycome of serum from the same patients and healthy individuals. This study highlighted significant changes in the glycosylation of ascitic proteins such as a decrease in high-mannose structures and an increase in branching, sialylation, and fucosylation. These alterations suggest crucial roles in the pathological mechanisms of ovarian cancer, opening new perspectives for diagnostics and therapies based on specific biomarkers.^77^

Additionally, CP can play an important role in the stratification of cancer patients.^78^ Studies such as the one by Krochmal et al. (2019) have demonstrated the usefulness of this approach in the stratification of bladder cancer, which has significant implications for choosing the most appropriate treatment.^78^

In the context of treatment monitoring, peptidic biomarkers have been discovered that can assess the efficacy of oncological treatments. Using a combination of nanoporous silica chips and MALDI-TOF, three peptides in plasma were identified as capable of distinguishing rectal cancer patients who benefited from presurgical chemotherapy from those who did not. This method achieved a high sensitivity of approximately 91% and a specificity of around 76%, resulting in an overall accuracy of about 86%.^69^

CP has great potential for applications in oncology, but it faces challenges such as the need for standardization in sample collection and analysis methods, as pointed out by He et al. (2022).

### Identification of Biomarkers in Acute Leukemias through Clinical Peptidomics

The uncontrolled proliferation of progenitor cells in both ALL and AML disrupts normal blood cell production, making serum and plasma valuable sources of biomarkers. These biomarkers can indicate the degree of disease progression, guide personalized therapeutic strategies, and facilitate early diagnosis.

Recent advancements in MS methodologies have made it possible to characterize peptides with greater precision across various biological samples. The progress in extraction techniques, MS, and bioinformatics, as referenced in recent oncology research, is playing a significant role in advancing CP. These developments offer new perspectives for understanding AL.

One study by Song et al. (2013a) investigated peptidomic profiles in the serum of AL patients, aiming to identify biomarkers for detecting minimal residual disease (MRD) and predicting patient prognosis (Table 1). The study utilized MS to analyze serum samples from AL patients and a control group without the disease. Key findings included the identification of a specific ion (*m*/*z* 4625) that was altered in AL patients, which effectively distinguished between AL patients and the control group with high sensitivity and specificity. Additionally, it was observed that the intensity of this peak was significantly related to AL prognosis, with a higher intensity seen in the relapse group compared to those in remission. The *m*/*z* 4625 peptide was subsequently identified via nano-LC-ESI-MS/MS as a fragment of SERPINA3, an acute-phase protein associated with inflammation and apoptosis.^61^

**Table 1:** Identified Peptides as Potential Biomarkers in Acute Leukemia and Their Clinical Implications

Author	*m*/*z*^g^ (Da)	Identified Peptide	Potential Function	Clinical Implications
Bai et al. (2013)	7762.87	PF4^e^	Platelet function	Diagnosis and monitoring of AML.^a^
Bai et al. (2013)	4089.7	Fibrinogen α	Coagulation	Diagnosis and monitoring of AML.^a^
Bai et al. (2013)	3216.57	UBA1^b^	Cellular protein degradation	Biomarker for MRD^c^ in AML.^a^
Song et al. (2013a)	4625	SERPINA3 fragment	Apoptosis; invasion and metastasis	Diagnosis of acute leukemia and MRD assessment.
Song et al. (2013b)	4468	SERPINA3 fragment	Apoptosis; invasion and metastasis	Diagnosis of acute leukemia and MRD^c^ assessment.
Bai et al. (2014)	2661.27	Fibrinogen α chain precursor	Coagulation	Monitoring MRD^c^ and predicting relapse in ALL.^d^
Bai et al. (2014)	2991.46	GSTP1^e^	Drug metabolism and resistance	Monitoring MRD^c^ and predicting relapse in ALL.^d^
Bai et al. (2014)	3443.92	Fibrinogen α chain precursor	Coagulation	Monitoring MRD^c^ and predicting relapse in ALL.^d^
Bai et al. (2014)	7764.29	PF4	Platelet function	Monitoring MRD^c^ and predicting relapse in ALL.^d^
Bai et al. (2014)	9288.31	CTAP-III^f^	Tumor angiogenesis	Monitoring MRD^c^ and predicting relapse in ALL.^d^

a AML - Acute Myeloid Leukemia; PF4 - Platelet Factor 4.

b UBA1 - Ubiquitin-Like Modifier-Activating Enzyme 1.

c MRD - Minimal Residual Disease.

d ALL - Acute Lymphoblastic Leukemia.

e GSTP1 - Glutathione S-transferase P1.

f CTAP-III - Connective Tissue Activating Peptide III.

g *m*/*z*: Mass-to-Charge Ratio.

Further analysis by Song et al. (2013b) examined serum from 105 AL patients and revealed the peptide *m*/*z* 4468, also a fragment of SERPINA3. The analysis indicated that this peptide may correlate with patient prognosis, suggesting that serum peptides can reflect the presence of AL and be valuable for monitoring MRD. The study highlighted the potential of these biomarkers for more accurate and early disease detection, which can aid in personalizing treatment strategies, thereby improving therapeutic success and patient survival.^79^

Bai et al. (2013) expanded on these findings by investigating the serum peptidomic profiles of newly diagnosed AML patients compared to a control group. The MS analysis revealed 47 significantly different peptide peaks were found. Among these peptides, ubiquitin-activating enzyme 1 (UBA1), the alpha chain of fibrinogen, and platelet factor 4 (PF4) emerged as promising candidates for monitoring MRD.^80^

A follow-up study by Bai et al. (2014) focused on potential serum peptidome-based biomarkers for MRD monitoring in adults with ALL. The research highlighted several peptides in the serum, including fragments of the alpha chain of fibrinogen, glutathione S-transferase P1, isoform 1 of the alpha chain fibrinogen precursor 1, and platelet factor 4. These molecules were suggested as useful for improving the detection and monitoring of the disease in its early stages. These findings are significant because they offer a new perspective on the management of ALL, allowing for more personalized interventions.^81^

Studies on the application of peptidomics in AL remain limited, with most research to date focusing primarily on serum samples. To date, no investigations have been identified that utilize CP in plasma samples from AL patients, highlighting the need to expand research efforts to include plasma samples and more diverse populations. This expansion is essential to enhance the applicability and generalizability of the results.^3^

Despite these limitations, the technique has shown promising potential. Preliminary studies have identified biomarkers through peptidomics, although their ability to differentiate between AML and acute ALL has yet to be corroborated. Nevertheless, peptidomics offers significant potential for differential diagnosis and the stratification of leukemia subtypes, enabling more accurate diagnoses with direct implications for the prognosis and the selection of personalized therapeutic interventions. The lack of robust evidence to date underscores the need for additional studies and rigorous clinical validations to effectively confirm the identification of specific biomarkers for each leukemia subtype.

Although the potential of CP is evident, its implementation in clinical practice still faces significant challenges, such as the lack of standardized guidelines and the need for consistent methodological validations. So far, studies have involved small cohorts and exhibited considerable methodological variability, compromising the reproducibility of the results obtained. Therefore, larger and more consistent studies are essential to enable the practical application of peptidomics in clinical settings.

On the other hand, the implementation of CP in clinical practice is already a tangible possibility. MALDI-TOF is widely applied in clinical practice, particularly within clinical microbiology, where its use has been well established. Its primary advantages include rapid identification capabilities and the low cost of consumables, which have significantly benefited microbiology laboratories.^82^ The adaptation of this technology for the detection of peptidomic biomarkers in AL offers significant advantages, allowing for its integration into laboratory routines without requiring major structural modifications.

Additionally, the development of methodologies that allow for the direct analysis of plasma or serum, without complex steps such as protein digestion, streamlines the process and makes it more accessible. This simplification facilitates the incorporation of peptidomics into clinical settings, reducing both the time and costs involved without compromising the quality of results.^83^ The precise identification of peptidomic biomarkers can enhance the effectiveness of treatments by avoiding ineffective interventions and minimizing adverse effects.

Although CP is not yet widely used as a routine diagnostic tool, advancements in simplifying analytical processes and the adoption of established technologies, such as MALDI-TOF, indicate that its clinical application is drawing closer. These developments position peptidomics as a cost-effective approach with great potential to compete with other well-established diagnostic technologies.

Moreover, studies applying CP in acute leukemias have shown its value in advancing a more thorough understanding of the disease and enhancing therapeutic approaches. The identification of specific peptides as biomarkers for prognosis, MRD, and treatment responses offers new perspectives for more accurate diagnoses and personalized therapies. Thus, CP not only enhances our understanding of the complexity of AL but also contributes to improving patients’ quality of life. Therefore, this technique has the potential to become an essential tool in the evolution of hematologic oncology.

## Conclusion

In conclusion, CP holds great potential to improve the diagnosis, prognosis, and treatment of AL, particularly through the identification of specific biomarkers. Although studies involving patients with AL have so far focused on serum samples, with no investigations conducted on plasma, research has already shown that CP can enhance both the understanding and treatment of the disease, with a direct impact on patients’ quality of life.

However, the technique still faces challenges, such as the need for robust clinical validation, methodological standardization, and expansion of studies to include plasma samples and more diverse populations. Technologies like MALDI-TOF, which are widely used in clinical microbiology, have simplified the implementation of CP, making it a cost-effective approach that is increasingly closer to clinical practice. Additional studies will be essential to solidify the use of CP and ensure its applicability in personalized therapies, ultimately improving clinical outcomes for AL patients.

Thus, clinical PC has the potential to establish itself as an essential tool in the advancement of hematologic oncology, not only by deepening our understanding of the complexity of acute leukemias but also by contributing to more effective interventions.
